# Case report: direct suture repair of inferior vena cava to rescue a stab patient with hepatic and caval injury through left hepatectomy and total vascular exclusion

**DOI:** 10.1186/s40792-021-01139-1

**Published:** 2021-02-23

**Authors:** Reina Hirooka, Kyoji Ito, Nobuyuki Takemura, Fuminori Mihara, Norihiro Kokudo

**Affiliations:** Hepato-Biliary-Pancreatic Surgery Division, Department of Surgery, National Center for Global Health and Medicine (NCGM), 1-21-1 Toyama, Shinjuku-ku, Tokyo, 162-8655 Japan

**Keywords:** Liver trauma, Damage control surgery, Retrohepatic inferior vena cava injury, Total vascular exclusion

## Abstract

**Background:**

The mortality of abdominal vena caval injuries is as high as 50–80%. Yet, there were few reports on how to repair injured inferior vena cava (IVC). This report presents a method of vena caval repair in a case of penetrating retrohepatic IVC injury, requiring hepatic resection and total vascular exclusion (TVE).

**Case presentation:**

The patient was a 20-year-old man with a stab wound in the epigastrium. An emergency laparotomy was performed in the emergency room, and a stab incision on the left liver was detected. As the Pringle’s maneuver did not reduce bleeding, hepatic vein injury was suspected, and left hemihepatectomy was performed to confirm the bleeding point. After the hepatectomy, laceration was still evident deeper into the resection, and IVC injury was suspected. The bleeding was temporarily controlled by tentative hepatorrhaphy and gauze packing, and the initial damage control surgery was terminated. Definitive surgery was performed on the third postoperative day. The lacerated point was observed under TVE, and the laceration penetrated the retrohepatic IVC through its posterior wall. The slit of the posterior wall was sutured first, followed by suturing of the anterior wall of the IVC. Finally, the lacerated liver was closed with hepatorrhaphy. TVE was removed, and the massive bleeding was successfully controlled.

**Conclusion:**

In severe liver injuries involving the retrohepatic IVC, hepatic resection and TVE may be useful for ensuring an optimized surgical field for repairing the injured IVC.

## Background

The liver is one of the most frequently damaged organs in abdominal trauma, and when the inferior vena cava (IVC) is involved, mortality is high (50–80%) [1–3]. In liver trauma, while nonoperative management is preferred for stable patients, laparotomy should be performed for unstable patients, especially for injuries greater than grade III as measured by the American Association for the Surgery of Trauma–Organ Injury Scale (AAST-OIS). The three most frequently used damage control surgical techniques for liver trauma are: (1) perihepatic packing, (2) hepatorrhaphy with or without hepatotomy, and (3) debridement hepatectomy [4]. Although different operative methods for major vessel injuries and associated mortality in liver trauma have been reported in previous studies, there have been few reports that explain the details of IVC suture repair. This report aimed to present a case of penetrating retrohepatic IVC injury requiring liver parenchymal resection and total vascular exclusion (TVE), including the step-by-step procedure of how the IVC was sutured and repaired.

## Case report

A previously healthy 20-year-old man was admitted to our emergency department after being stabbed by a young female during a violent fight between the two. The patient presented with massive bleeding from a wound in the epigastrium. Abdominal ultrasonography revealed an abundance of free fluid in Morrison’s pouch and the perisplenic space. Hand compression of the wound and aggressive fluid therapy were performed simultaneously. With only a slight hemodynamic improvement, an emergency laparotomy in the emergency room (ER) was immediately initiated.

**Fig. 1:**
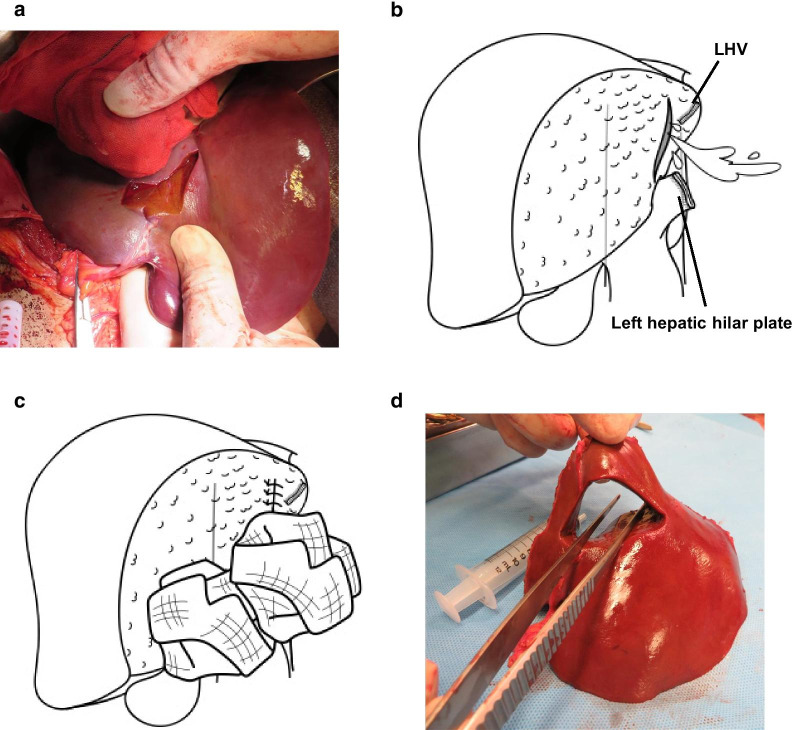
Operative findings in the damage control surgery. **a** A stab incision on the surface of the left liver. **b** The laceration of the liver parenchyma reached deeper into the resection surface after left hemihepatectomy, and bleeding persistently continued from the laceration. LHV, left hepatic vein. **c** Bleeding was controlled even suturing of the lacerated liver parenchyma and gauze packing. **d** In the resected specimen of the left liver, the laceration penetrated from the liver surface to the resection surface

Laparotomy was conducted through an abdominal midline incision, and there was massive hematoma in the abdominal cavity. The stab wound was identified on the surface of the left liver, where the bleeding was profuse and continuous (Fig. 1a). The skin incision was extended into an inverted L-shape to adequately expose the whole liver. Since clamping of the hepatoduodenal ligament using the Pringle’s maneuver did not reduce bleeding, a deep liver laceration involving a hepatic vein or IVC injury was suspected. Neither hand compression nor gauze packing controlled the bleeding, and therefore, a left hepatectomy was performed to adequately expose the injured vessels. The left hepatic vein was ligated and divided, the left hepatic hilar plate was divided using a stapler, and the left hepatectomy was completed. However, the laceration of the liver parenchyma reached deeper into the resection surface, and persistent bleeding continued from the laceration (Fig. 1b). Suturing of the lacerated liver parenchyma was attempted. However, bleeding was still unable to be controlled (Fig. 1c). Fortunately, temporal hemostasis was achieved by gauze packing probably because hemostasis from the injured peripheral left hepatic vein was obtained and gauze compression close to the bleeding point was effective. The initial procedure was terminated as a damage control surgery, with consideration for the patient’s coagulopathy and physiological instability due to the massive blood transfusion required during the procedure. In total, 28 units of red blood cells, 32 units of fresh frozen plasma, and 30 units of platelet concentrate were transfused. The operative time was 97 min. The blood loss was approximately 2000 g. In the resected specimen of the left liver, there was a laceration penetrated from the liver surface to the resection surface (Fig. 1d). Postoperative course after the damage control surgery was favorable. There was no sign of re-bleeding in the abdomen and his vital signs were stable. Since IVC injury was implied considering the massive bleeding from the laceration, we made an arrangement of surgeons, anesthesiologists, and blood transfusion to fully prepare a second-look surgery.

**Fig. 2:**
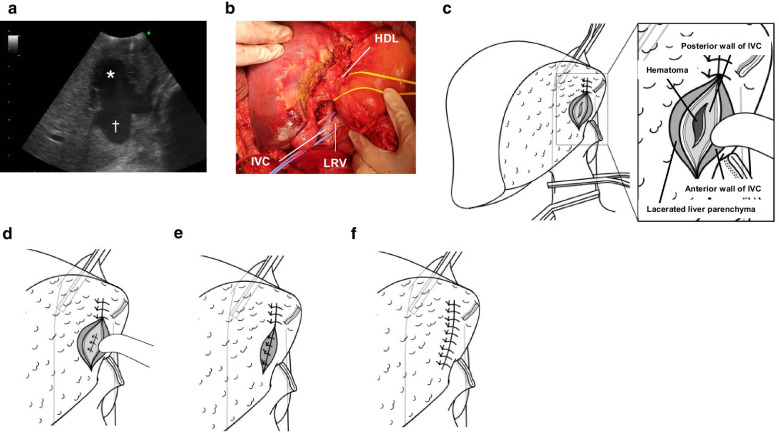
Operative findings in the definitive surgery. **a** Intraoperative ultrasonography showing a free space (*) in front of the IVC (†), indicating IVC injury. **b** Taping of the HDL and major veins. HDL, hepatoduodenal ligament; LRV, left renal vein. **c** Observation of the lacerated IVC. The laceration penetrated the retrohepatic IVC through its posterior wall. Hematoma was observed behind the posterior wall of theretrohepatic IVC. **d** The posterior wall of IVC was sutured. **e** The anterior wall of IVC was sutured together with the lacerated hepatic parenchyma. **f** Liver parenchyma was sutured, and hemostasis was finally obtained

The second-look surgery was then performed on the third operative day in the operating room. Upon opening the wound, there was no remarkable bleeding. However, severe bleeding was observed after gauze packing was removed from the resection surface of the liver. Intraoperative ultrasonography confirmed free fluid anterior to the retrohepatic IVC, suggesting IVC injury and pooling of a hematoma (Fig. 2a). TVE was planned for vascular isolation. The infrahepatic IVC was taped superior to the left renal vein (Fig. 2b), and the suprahepatic IVC was exposed. TVE was applied, and the lacerated site was observed (Fig. 2c). Evidently, the laceration reached and penetrated the retrohepatic IVC. To repair the IVC, the slit of the posterior wall was sutured first (Fig. 2d). Subsequently, the anterior wall of the IVC was sutured together with liver parenchyma (Fig. 2e). Finally, the lacerated liver parenchyma was sutured and repaired for closure (Fig. 2f). Hemostasis was confirmed after the release of TVE. The duration of TVE was 27 min. The operative time was 161 min, and blood loss was 3721 g. In total, 22 units of red blood cells and 4 units of fresh frozen plasma were transfused.

During the postoperative course, the patient developed minor bile leakage, which was relieved conservatively. The patient was discharged on postoperative day 35. The patient was successfully rehabilitated after discharge and returned work.

## Discussion

The liver is the most frequently damaged organ in abdominal trauma [4], and the most widely used classification system for liver injury is the AAST-OIS [5]. Grade I or II injuries are relatively inconsequential, such as minor capsular tears, that do not require operative treatments. Grade III includes vascular injury associated with a hematoma or parenchymal laceration. Injuries greater than grade III are severe and often require operative management. Grade IV injuries are characterized by major parenchymal disruption, and Grade V injuries are the most severe, involving juxtahepatic venous injuries. High-grade liver injuries are associated with poorer prognosis, and thus, an appropriate management strategy should be developed in each emergency hospital [6]. Lin et al. [7] showed their treatment algorism for liver injury and presented 58 patients with blunt liver injury equal to or greater than AAST-OIS grade III. The patients with 14 grade V injuries were mainly managed with debridement hepatectomy and venous repair. Four patients underwent IVC repair and only one survived after IVC repair, although the precise method used in the IVC repair was not reported.

Injury to the retrohepatic vena cava accounts for only a small proportion of all liver injuries. However, it has been described as the most technically difficult and most deadly form of liver trauma [8]. Its mortality remains high, ranging between 50 and 80% [9]. The three major operative methods for hepatic veins or retrohepatic vena caval injuries include (a) direct suture repair of the bleeding vessels with or without vascular isolation, (b) lobar resection, and (c) intraparenchymal or perihepatic packing [8]. If hemorrhage control is not achieved by the major operative methods, then a definitive surgery to repair the hepatic vein or retrohepatic IVC using TVE, atrial-caval shunt, or bio pump, is required [10]. Of these, TVE might be especially useful, since it does not require extracorporeal circulation and can be performed in the surgical site.

In reported series, most institutions preferred direct exposure and suture repair of the hepatic vein or retrohepatic caval wounds, regardless of injury patterns [11]. In a large multicenter series, Cogbill et al. reported a mortality rate of 90% for patients with main hepatic vein or caval injuries in whom atriocaval shunting had been used [12]. Gao et al. reported 7 cases of retrohepatic IVC injury repair with hepatectomy by total vascular exclusion and the mortality rate was 77.8% (7/9) [10]. Others have reported similar horrendous results [2, 3]. In more recent years, a smaller retrospective study was conducted by Hansen and colleagues [13]. They investigated 47 patients with abdominal vena caval injuries and examined the type of repair performed with respect to the outcome. The repair methods included suturing, ligation, vein patch, and graft patch, and the most common repair of IVC injury was suturing (72%). Of the 47 patients, there were 9 cases of retrohepatic IVC injury with a mortality rate of 66% (6/9).

Although different operative methods and associated mortality for major venous injuries have been reported, there have been few reports focusing on the detailed procedure of vena caval suture repair. In the present report, we present a hemodynamically unstable patient with grade V liver injury who required emergency laparotomy at the ER. The laceration reached the retrohepatic IVC, and hemostasis was achieved by suturing the injured IVC following left hemihepatectomy and TVE. In a deep stab wound to the liver suspected of retrohepatic IVC injury, it is essential to obtain an adequate operation field to thoroughly observe the extent of injury. As there are various patterns and degrees of seriousness of IVC injuries, different and individualized case-oriented approaches to repair the injured IVC must be considered, and these methods should be more precisely investigated in future studies.

## Conclusion

In severe liver injuries involving the retrohepatic IVC, hepatic resection and TVE may be useful for ensuring an optimized surgical field for repairing the injured IVC.

## Data Availability

The datasets analyzed during the current study are not publicly available, because they contain information that could compromise the privacy of research participants but are available from the corresponding author on reasonable request.

## Abbreviations

AAST-OIS: The American Association for the Surgery of Trauma-Organ Injury Scale
IVC: Inferior vena cava
TVE: Total vascular exclusion
